# Repeated Changes to the Gravitational Field Negatively Affect the Serum Concentration of Select Growth Factors and Cytokines

**DOI:** 10.3389/fphys.2019.00402

**Published:** 2019-04-17

**Authors:** Ulrik Stervbo, Toralf Roch, Timm H. Westhoff, Ludmyla Gayova, Andrii Kurchenko, Felix S. Seibert, Nina Babel

**Affiliations:** ^1^Center for Translational Medicine, Medical Department I, Marien Hospital Herne, University Hospitals of the Ruhr-University of Bochum, Herne, Germany; ^2^Charité – Universitätsmedizin Berlin, Corporate Member of Freie Universität Berlin, Humboldt-Universität zu Berlin, Berlin Institute of Health, Berlin-Brandenburg Center for Regenerative Therapies, Berlin, Germany; ^3^Department of Bioorganic and Biological Chemistry, Bogomolets National Medical University, Kyiv, Ukraine

**Keywords:** microgravity, hypergravity, parabolic flights, gravitational stress, bone remodeling, pro-inflammatory cytokine

## Abstract

Space flights, some physical activities, and extreme sports can greatly alter the gravitational forces experienced by the body. Being a deviation from the constant pull of Earth, these alterations can be considered gravitational stress and have the potential to affect physiological processes. Physical cues play a vital role in the homeostasis and function of the immune system. The effect of recurrent alterations of the gravitational pull on the levels of soluble mediator such as cytokines is unknown. Parabolic flights provide a controlled environment and make these a suitable model to study the effects of gravitational stress. Utilizing this model, we evaluated the effects of short-term gravitational stress on serum concentration of cytokines and other soluble mediators. Blood was taken from 12 healthy volunteers immediately before the first parabola and immediately after the last. Samples taken on the ground at corresponding time points the day before were used to control for circadian effects. A wide range of soluble mediators was analyzed using a multiplex bead assay. We found that the rate-change of eight molecules was significantly affected by the parabolic flight. Among other functions, these molecules, EGF, PDGF-AA, PDGF-BB, HGF, IP-10, Eotaxin (CCL11), TARC, and Angiopoietin-2, can be associated with bone remodeling and immune activation. It is therefore possible that gravitational stress can have clinically relevant impact on the control of a wide range of physiological processes.

## Introduction

All organisms on earth have evolved under a constant gravitational acceleration of 1 *g*. Space flight is a clear deviation from this, but also physical exercises and extreme sports, such as some amusement rides, skydiving, bungee jumping, or wing-suit flying, can change the experienced gravitational force and elicit acute stress (van Westerloo et al., 2011; Gomez and Rao, 2016; Liu et al., 2016). The changes range from microgravity at near weightlessness to hypergravity. In all cases, rapid and repeated changes can be experienced. These gravitational alterations are stressful by nature, and can have adverse effects such as impaired tissue, organ, and immune functionalities (Guéguinou et al., 2009; Crucian et al., 2018).

Given the omnipresence of the gravitational pull of Earth, it is difficult to model the effect of microgravity in a laboratory. This is in contrast to hypergravity, which can be achieved through centrifugation. Nonetheless, several *in vitro* and *in vivo* models, which mimic the physiological changes during decreased gravity, have been established (Clément, 2017). Parabolic flights have well defined periods of micro- and hypergravity and are therefore an attractive model to study different physiological responses to short-term gravitational stress (Le Bourg, 1999).

Assuming physiological responses to repeated changes of the gravitational field, we were wondering how this stress affects the serum concentration of soluble mediators such as cytokines and growth factors. We therefore collected serum from 12 healthy volunteers before and immediately after the parabolic flight maneuvers. The sera were analyzed for 51 mediators using multiplex bead assays and compared to sera collected one day before the flight at the same time points to control for circadian effects (Keller et al., 2009).

## Materials and Methods

### Study Population and Ethics Statement

Blood was obtained from 12 apparently healthy volunteers; 5 females (mean 24.2 years, range 21–29), 7 males (mean 29.1 years, range 21–43). The study protocol was approved by the ethical committee of the Ruhr-Universität Bochum (register-number 5158-14). Exclusion criteria were intake of any kind of medication on a regular basis and a history of cardiovascular diseases including hypertension, coronary heart disease, congestive heart failure, stroke, as well as immune disorders, including diabetes, asthma, or allergies.

All participants provided written informed consent prior inclusion in the study. Participants of parabolic flights usually receive scopolamine as kinetosis prophylaxis, which was refrained in this study in order to avoid any drug-related changes.

### Parabolic Flight and Blood Donation

**FIGURE 1:**
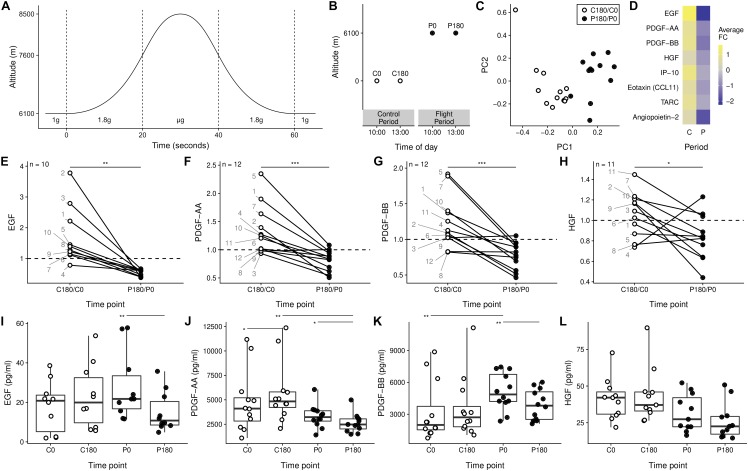
Soluble mediators are decreased after gravitational stress. **(A)** Single parabola with alteration in experienced gravitational acceleration. **(B)** Sampling scheme applied in the study – blood was taken before the first parabola (P0) and after the last (P180), both during 1 *g*. The day before, two samples were taken on the ground at time points of the corresponding in-flight samples (C0 and C180). **(C)** Principal component analysis of the change in concentration of all 51 measured molecules from t_180_ to t_0_. **(D)** Soluble mediators with significant fold change. **(E–H)** Soluble mediators with different time related changed between the control phase and the flight phase. The individual donors are annotated by each point for the C180/C0 ratio. **(I–L)** Individual samples of the data presented in panels **(E–H)**. ^∗^*p* < 0.05, ^∗∗^*p* < 0.01, and ^∗∗∗^*p* < 0.001.

An Airbus A310 performed a single successful test parabola and 30 regular parabolas in which phases of weightlessness were achieved. This microgravity phase of each parabola was flanked by a pull-up and a pull-out phase at about 1.8 *g* hypergravity (Figure 1A). Each phase lasted about 20 s. The parabolas were performed in six sets of five parabolas. Each set was separated by steady flight of 5 to 8 min. The steady flight in 1 *g* between two parabolas lasted for 2 min. The cabin conditions remained stable at all times with a pressure of 830 mbar, cabin luminosity of 800 lux and a temperature of 19–21°C with a humidity of 15%. All participants were at rest throughout the flight.

The participants had no prior experience with parabolic flights. Each participant flew just once, and was equipped with an intravenous cannula, from which the blood samples were obtained during the flight. Blood was collected in S-Monovette Z-Gel tubes (Sarstedt, Germany). Serum was obtained by centrifugation at 2500 *g*, 4 min, room temperature immediately after arrival of the plane. The decanted serum was stored at -80°C until use.

### Assessment of Serum Concentration of Soluble Mediators

Fifty one soluble mediators were assessed using four different LEGENDplex panels (BioLegend, Germany), covering IL-5, IL-13, IL-2, IL-6, IL-9, IL-10, IFN-γ, TNF-α, IL-17A, IL-17F, IL-4, IL-21, and IL-22 (Human Th Cytokine Panel), TSLP, IL-1α, IL-1β, GM-CSF, IFN-α, IL-23, IL-12p40, IL-12p70, IL-15, IL-18, IL-11, IL-27, and IL-33 (Human Cytokine Panel 2), IL-8, IP-10, Eotaxin, TARC, MCP-1, RANTES, MIP-1α, MIG, ENA-78, MIP-3α, GROα, I-TAC, and MIP-1β (Human Proinflammatory Chemokine Panel), and Angiopoietin-2, EGF, EPO, FGF-basic, G-CSF, GM-CSF, HGF, M-CSF, PDGF-AA, PDGF-BB, SCF, TGF-α, and VEGF (Human Growth Factor Panel). The samples were processed per manufacturer’s instructions. Briefly, 1:2 diluted serum samples were mixed with assay beads in a V-bottom microtiter plate at 500 rpm, at room temperature for 2 h. Detection antibodies were added, and incubated for 1 h at 500 rpm followed by addition of Streptavidin-PE detection antibody for 30 min at 500 rpm. Samples were acquired on a CytoFLEX flow cytometer (Beckman Coulter, Germany), and the concentration was extracted from the resulting fcs-files using beadplexr, version 0.1 (Stervbo et al., 2018b).

Inclusion of a mediator in the statistical analysis required a measurable concentration at all four time points in at least three donors. The analyzed mediators were occasionally below the standard range, leading to missing measurements for a few of the mediators and donors (Supplementary Figure 1).

### Statistical Analysis and Graphical Representation

Data were analyzed with R, version 3.5.1. Statistical comparison was performed using a repeated measure One Way ANOVA followed by Sidak’s multiple comparison test where appropriate. The box-whiskers plot indicates the 25, 50, and 75 percentile as well as range. ^∗^*p* < 0.05, ^∗∗^*p* < 0.01, and ^∗∗∗^*p* < 0.001 of the indicated comparisons. Each point signifies a donor.

## Results

Blood samples were taken circa 30 min after takeoff prior to the first parabolic maneuver (P0) and 3 h later after the last parabola (P180) before the landing procedure initiation (Figure 1B). As control, blood was taken at corresponding time points on the ground 24 h before the parabolic flight (C0 and C180). Thus, the time span from C0 to C180 is referred to as control period and P0 to P180 as parabolic flight period (Figure 1B).

To exclude the circadian influence we focused our analysis on the rate-change of inflammatory mediators in the control (C180/C0) and in the parabolic flight period (P180/P0). Applying the dimension reduction method principle component analysis (PCA), we found a clear separation of the rate-change in the control period compared to the flight period in the first principal component alone (Figure 1C). This separation was driven by a set of eight soluble mediators with a significant average fold change (Figure 1D). In addition, we found no correlation between the rate-change of cortisol to the rate-change of the soluble mediators (Supplementary Figure 2). Though slightly less pronounced in the females, we observed a similar reduction in soluble mediators for both genders (Supplementary Figure 3). Our data thus indicate that repeated changes to the gravitational field cause alteration to the serum concentration of different soluble mediators.

The ratio of the growth factors EGF, PDGF-AA, PDGF-BB, and HGF decreased during the flight period compared to the control period (Figure 1E–H). This change in ratio was not only due to particular differences at the start of the flight compared to the control samples (Figure 1I–L). Our data therefore support the possibility that gravitational stress affects the serum concentration of factors, which are involved in numeral biological processes.

**FIGURE 2:**
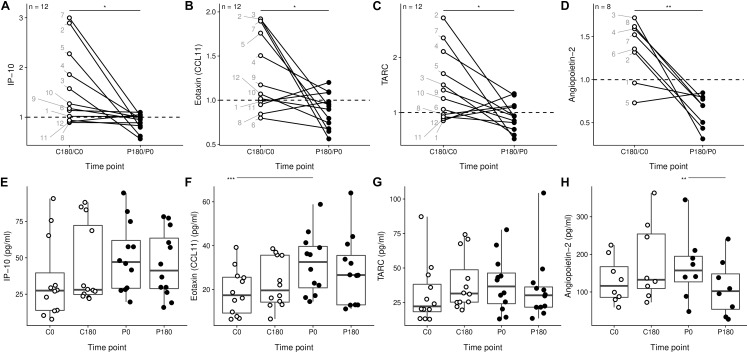
Gravitational stress results in a decrease of soluble mediators with immune modulatory capacities. **(A–D)** Soluble mediators with different time related changes between the control phase and the flight phase. The individual donors are annotated by each point for the C180/C0 ratio. **(E–H)** Individual samples of the data presented in panels **(A–D)**. ^∗^*p* < 0.05, ^∗∗^*p* < 0.01, and ^∗∗∗^*p* < 0.001.

Soluble mediators with immune modulatory capacities such as IP-10, Eotaxin (CCL11), TARC, and Angiopoietin-2 have an average ratio for the flight period which is less than one, and significantly lower than the ratio for the control period (Figure 2A–D). Since the decreased rate-change was not due to a particularly skewed time point (Figure 2E–H), we conclude that the parabolic flight is associated with a decrease in serum levels of inflammatory mediators.

To elucidate possible interactions between the identified mediators we utilized the Reactome Pathway Knowledgebase (Fabregat et al., 2018). The database was queried with the UniProt IDs: P01133 (EGF), PDGF1 (PDGFA), P01127 (PDGFB), P51671 (Eotaxin CCL11), P14210 (HGF), P02778 (IP-10), Q92583 (TARC), and O15123 (Angiopoietin-2). We found an effect of the identified mediators on the JAK/STAT signaling axis as well as PI3K/Akt and RAF/MAP signaling axis (data not shown). This observation suggests cytokine interaction and their important role in interleukin signaling and general signal transduction.

## Discussion

Gravitational stress can be experienced through different physical activities and – as with stress in general – it can potentially affect several biological processes. Still, the mechanistic impact of short and of long-term gravitational stress is not known. We report here on eight soluble factors that decrease during parabolic flights with potential biological effects (Supplementary Table 1). In addition to the primary roles, EGF, PDGF-AA, PDGF-BB, and HGF have all been associated with bone homeostasis and bone remodeling (Kratchmarova et al., 2005; Nakamura and Mizuno, 2010). IP-10, Eotaxin (CCL11), and TARC are known to play a role in immune cell activation though their roles as chemoattractant (Pease and Horuk, 2009a,b; Karin and Razon, 2018). The vascular growth factor Angiopoietin-2 might also be associated with immune regulation, as it has been demonstrated that the ratio of Angiopoietin-2 to Angiopoietin-1 is associated with poor clinical outcome of early sepsis (Fang et al., 2015). The decrease of Eotaxin is in line with previous observations after 60 day bed rest (Hoff et al., 2015) and associates with our previous observation of fewer circulating eosinophils (Stervbo et al., 2018a).

In line with our observations, EGF has been reported reduced after physical exercise (Accattato et al., 2017). The platelet-derived growth factors PDGF-AA and PDGF-BB have been have been reported to have a protective effect in oxidative stress (Zheng et al., 2010, 2013). An association with PDGF expression and sheer stress has further been reported (Resnick et al., 1997; Wilson et al., 1998). However, no effect of changes to peripheral systolic blood pressure could be observed in our cohort (Seibert et al., 2018). In the context of gravitational stress, TARC is particularly interesting, because this molecule has been suggested as a candidate biomarker for posttraumatic stress disorder in males (Dalgard et al., 2017).

It has previously been observed that long term space flight can lead to changes in the plasma concentration of the growth factors TPO and VEGF and of the pro-inflammatory cytokines TNF-α and IL-8 (Crucian et al., 2014). The latter is in line with observations, that long term space flight depress the cytokine producing capabilities of T-cells after mitogenic stimulation (Crucian et al., 2015). Our results reveal no change to these factors, and we ascribe this contrast to the repeated alteration between hyper- and hypogravity experiences on a parabola flight compared to the constant microgravity during long term space flights. Acute stress caused by bungee jump has likewise been shown to cause a decrease in TNF-α, IL-8, and IL-10 (van Westerloo et al., 2011). The contrast to our study is probably found in the different approach taken to assess the stress induced effect on cytokines. Future studies will need to include measurement with and without *ex vivo* LPS stimulation to assess the full effect of stress on cytokines.

The pathway analysis revealed that the immediate effect of the eight identified soluble mediators involved the JAK/STAT axis, the PI3K/Akt axis, and the RAF/MAP axis. Collectively these pathways indicate a general response to cytokines and growth factors as exemplified by EGR and IL-3 signaling (Steelman et al., 2004; Di Domenico and Giordano, 2017). The cellular effect of these pathways range from apoptosis to cell survival, differentiation, and proliferation. Thus, additional studies are needed to elucidate the intermediate and long-term effect of these affected pathways.

The levels of the factors reported here are all within the ranges found in the literature for healthy individuals and potential biological effects of the gravitational stress induced alteration have to be analyzed in future studies (Ayache et al., 2006; Kim et al., 2011; Yu et al., 2015). However, not all mediators could be found at every time point, but measured levels were generally well within the assay standard range. Additionally, we observed no difference between C0 and C180 indicating that any change in mediator concentration during the parabolic flight, is due to the gravitational stress and not a circadian effect. For three mediators (EGF, PDGF-BB, and Eotaxin) we did observe a significant difference between C0 and P0, but attribute this to the anticipation and excitement of approaching the parabolic flight, which is in agreement with slightly elevated cortisol levels at P0 (Stervbo et al., 2018a).

We found for all eight mediators that the P180 levels were smaller than the P0 levels, and that the C0 levels were larger than the C180 level. This is a surprising finding, as it indicates that the molecules are removed from circulation after binding to their appropriate receptors. Consequently, this could point to an increase in receptor expression, and it will be interesting to see the interplay between soluble mediators and their receptors during and after gravitational stress. Alternatively, the decrease could be attributed to a decrease in the secreting cells, or the secretory function of these cells. When analyzing the response of females and males separately, we see a stronger effect in the males for some mediators compared to the females. We expect this to be due the slightly smaller female subgroup and general loss of power due to the sub-setting. It will be important for future studies to keep this aspect in mind.

The long-term effects of gravitational stress were not addressed in the present study. The participants remained apparently healthy after the flight and the impact of the alterations of the eight soluble mediators remains speculative. Though this pose some limitations on the conclusions which can be drawn, the data presented here nonetheless demonstrate that repeated changes to the gravitational acceleration can induce changes to circulating cytokines. Future studies should consider the potential effects on bone and immune homeostasis. This could be of particular relevance to the pilots and staff of the parabolic flights, fighter pilots, and others who are regularly subjected to gravitational stress.

## Ethics Statement

This study was carried out in accordance with the recommendations of the ethical committee of the Ruhr-Universität Bochum with written informed consent from all subjects. All subjects gave written informed consent in accordance with the Declaration of Helsinki. The protocol was approved by the ethical committee of the Ruhr-Universität Bochum (Register-Number 5158–14).

## Author Contributions

US, FS, LG, and AK performed the experiments. US and TR analyzed the data, created the figures, and drafted the manuscript. TW, FS, and NB designed the study. All authors revised the manuscript and approved its final version.

## Conflict of Interest Statement

The authors declare that the research was conducted in the absence of any commercial or financial relationships that could be construed as a potential conflict of interest.

## References

[B1] Accattato F. Greco M. Pullano S. A. Carè I. Fiorillo A. S. Pujia A. (2017). Effects of acute physical exercise on oxidative stress and inflammatory status in young, sedentary obese subjects. *PLoS One* 12:e0178900. 10.1371/journal.pone.0178900 28582461 PMC5459463

[B2] Ayache S. Panelli M. C. Byrne K. M. Slezak S. Leitman S. F. Marincola F. M. (2006). Comparison of proteomic profiles of serum, plasma, and modified media supplements used for cell culture and expansion. *J. Transl. Med.* 4:40. 10.1186/1479-5876-4-40 17020621 PMC1601968

[B3] Clément G. (2017). International roadmap for artificial gravity research. *NPJ Microgravity* 3:29. 10.1038/s41526-017-0034-8 29184903 PMC5701204

[B4] Crucian B. Stowe R. P. Mehta S. Quiriarte H. Pierson D. Sams C. (2015). Alterations in adaptive immunity persist during long-duration spaceflight. *NPJ Microgravity* 1:15013. 10.1038/npjmgrav.2015.13 28725716 PMC5515498

[B5] Crucian B. E. Choukèr A. Simpson R. J. Mehta S. Marshall G. Smith S. M. (2018). Immune system dysregulation during spaceflight: potential countermeasures for deep space exploration missions. *Front. Immunol.* 9:1437. 10.3389/fimmu.2018.01437 30018614 PMC6038331

[B6] Crucian B. E. Zwart S. R. Mehta S. Uchakin P. Quiriarte H. D. Pierson D. (2014). Plasma cytokine concentrations indicate that in vivo hormonal regulation of immunity is altered during long-duration spaceflight. *J. Interferon Cytokine Res.* 34 778–786. 10.1089/jir.2013.0129 24702175 PMC4186776

[B7] Dalgard C. Eidelman O. Jozwik C. Olsen C. H. Srivastava M. Biswas R. (2017). The MCP-4/MCP-1 ratio in plasma is a candidate circadian biomarker for chronic post-traumatic stress disorder. *Transl. Psychiatry* 7:e1025. 10.1038/tp.2016.285 28170001 PMC5438024

[B8] Di Domenico M. Giordano A. (2017). Signal transduction growth factors: the effective governance of transcription and cellular adhesion in cancer invasion. *Oncotarget* 8 36869–36884. 10.18632/oncotarget.16300 28415812 PMC5482705

[B9] Fabregat A. Jupe S. Matthews L. Sidiropoulos K. Gillespie M. Garapati P. (2018). The reactome pathway knowledgebase. *Nucleic Acids Res.* 46 D649–D655. 10.1093/nar/gkx1132 29145629 PMC5753187

[B10] Fang Y. Li C. Shao R. Yu H. Zhang Q. Zhao L. (2015). Prognostic significance of the angiopoietin-2/angiopoietin-1 and angiopoietin-1/Tie-2 ratios for early sepsis in an emergency department. *Crit. Care* 19:367. 10.1186/s13054-015-1075-6 26463042 PMC4604731

[B11] Gomez A. T. Rao A. (2016). Adventure and extreme sports. *Med. Clin. North Am.* 100 371–391. 10.1016/j.mcna.2015.09.009 26900120

[B12] Guéguinou N. Huin-Schohn C. Bascove M. Bueb J.-L. Tschirhart E. Legrand-Frossi C. (2009). Could spaceflight-associated immune system weakening preclude the expansion of human presence beyond Earth’s orbit? *J. Leukoc. Biol.* 86 1027–1038. 10.1189/jlb.0309167 19690292

[B13] Hoff P. Belavý D. L. Huscher D. Lang A. Hahne M. Kuhlmey A.-K. (2015). Effects of 60-day bed rest with and without exercise on cellular and humoral immunological parameters. *Cell. Mol. Immunol.* 12 483–492. 10.1038/cmi.2014.106 25382740 PMC4496533

[B14] Karin N. Razon H. (2018). Chemokines beyond chemo-attraction: CXCL10 and its significant role in cancer and autoimmunity. *Cytokine* 109 24–28. 10.1016/j.cyto.2018.02.012 29449068

[B15] Keller M. Mazuch J. Abraham U. Eom G. D. Herzog E. D. Volk H.-D. (2009). A circadian clock in macrophages controls inflammatory immune responses. *Proc. Natl. Acad. Sci. U.S.A.* 106 21407–21412. 10.1073/pnas.0906361106 19955445 PMC2795539

[B16] Kim H. O. Kim H.-S. Youn J.-C. Shin E.-C. Park S. (2011). Serum cytokine profiles in healthy young and elderly population assessed using multiplexed bead-based immunoassays. *J. Transl. Med.* 9:113. 10.1186/1479-5876-9-113 21774806 PMC3146842

[B17] Kratchmarova I. Blagoev B. Haack-Sorensen M. Kassem M. Mann M. (2005). Mechanism of divergent growth factor effects in mesenchymal stem cell differentiation. *Science* 308 1472–1477. 10.1126/science.1107627 15933201

[B18] Le Bourg E. (1999). A review of the effects of microgravity and of hypergravity on aging and longevity. *Exp. Gerontol.* 34 319–336. 10.1016/S0531-5565(99)00004-2 10433387

[B19] Liu T. Y. Wu Q. P. Sun B. Q. Han F. T. (2016). Microgravity Level Measurement of the Beijing Drop Tower Using a Sensitive Accelerometer. *Sci. Rep.* 6:31632. 10.1038/srep31632 27530726 PMC4987679

[B20] Nakamura T. Mizuno S. (2010). The discovery of hepatocyte growth factor (HGF) and its significance for cell biology, life sciences and clinical medicine. *Proc. Jpn. Acad. Ser. B Phys. Biol. Sci.* 86 588–610. 10.2183/pjab.86.588 20551596 PMC3081175

[B21] Pease J. E. Horuk R. (2009a). Chemokine receptor antagonists: part 1. *Expert Opin. Ther. Pat.* 19 39–58. 10.1517/13543770802641346 19441897

[B22] Pease J. E. Horuk R. (2009b). Chemokine receptor antagonists: part 2. *Expert Opin. Ther. Pat.* 19 199–221. 10.1517/13543770802641353 19441918

[B23] Resnick N. Yahav H. Khachigian L. M. Collins T. Anderson K. R. Dewey F. C. (1997). Endothelial gene regulation by laminar shear stress. *Adv. Exp. Med. Biol.* 430 155–164. 10.1007/978-1-4615-5959-7_13 9330726

[B24] Seibert F. S. Bernhard F. Stervbo U. Vairavanathan S. Bauer F. Rohn B. (2018). The effect of microgravity on central aortic blood pressure. *Am. J. Hypertens.* 31 1183–1189. 10.1093/ajh/hpy119 30052726

[B25] Steelman L. S. Pohnert S. C. Shelton J. G. Franklin R. A. Bertrand F. E. McCubrey J. A. (2004). JAK/STAT, Raf/MEK/ERK, PI3K/Akt and BCR-ABL in cell cycle progression and leukemogenesis. *Leukemia* 18 189–218. 10.1038/sj.leu.2403241 14737178

[B26] Stervbo U. Roch T. Kornprobst T. Sawitzki B. Grütz G. Wilhelm A. (2018a). Gravitational stress during parabolic flights reduces the number of circulating innate and adaptive leukocyte subsets in human blood. *PLoS One* 13:e0206272. 10.1371/journal.pone.0206272 30427865 PMC6235284

[B27] Stervbo U. Westhoff T. H. Babel N. (2018b). beadplexr: reproducible and automated analysis of multiplex bead assays. *PeerJ* 6:e5794. 10.7717/peerj.5794 30479885 PMC6241392

[B28] van Westerloo D. J. Choi G. Löwenberg E. C. Truijen J. de Vos A. F. Endert E. (2011). Acute stress elicited by bungee jumping suppresses human innate immunity^∗∗^. *Mol. Med.* 17:180. 10.2119/molmed.2010.00204 21203694 PMC3060977

[B29] Wilson E. Vives F. Collins T. Ives H. E. (1998). Strain-responsive regions in the platelet-derived growth factor-A gene promoter. *Hypertens. Dallas Tex* 31 170–175. 10.1161/01.HYP.31.1.170 9453298

[B30] Yu H. Wang Y. Yu Q. Zhang H. Ma W. Shang S. (2015). Significance of plasma hepatocyte growth factor in diagnosis of benign and malignant solitary pulmonary nodules. *Int. J. Clin. Exp. Pathol.* 8 2063–2067. 25973105 PMC4396333

[B31] Zheng L. Ishii Y. Tokunaga A. Hamashima T. Shen J. Zhao Q.-L. (2010). Neuroprotective effects of PDGF against oxidative stress and the signaling pathway involved. *J. Neurosci. Res.* 88 1273–1284. 10.1002/jnr.22302 19998489

[B32] Zheng L.-S. Ishii Y. Zhao Q.-L. Kondo T. Sasahara M. (2013). PDGF suppresses oxidative stress induced Ca2+ overload and calpain activation in neurons. *Oxid. Med. Cell. Longev.* 2013:367206. 10.1155/2013/367206 24454980 PMC3886591

