# Supplementary material for: Repeated Changes to the Gravitational Field Negatively Affect the Serum Concentration of Select Growth Factors and Cytokines
Source: Front Physiol. 2019 Apr 17;10:402. doi: 10.3389/fphys.2019.00402 (PMC6478750; doi:10.3389/fphys.2019.00402)
Supplement: Supplementary file 1 [file Data_Sheet_1.PDF]

Supplementary Material

Repeated Changes to the Gravitational Field Negatively Affect the Serum Concentration of Select Growth Factors and Cytokines

Ulrik Stervbo[1†], Toralf Roch[2†], Timm H. Westhoff[1], Ludmyla Gayova[3], Andrii Kurchenko[3], Felix S. Seibert[1¶], Nina Babel[1,2¶*]

[1]Center for Translational Medicine, Medical Department I, Marien Hospital Herne, University Hospital of the Ruhr-University Bochum, Herne, Germany
[2]Charité – Universitätsmedizin Berlin, corporate member of Freie Universität Berlin, Humboldt-Universität zu Berlin, and Berlin Institute of Health, Berlin-Brandenburg Center for Regenerative Therapies, Berlin, Germany
[3]Bogomolets National Medical University, Kyiv, Ukraine

*** Correspondence:** Nina Babel: Nina.babel@charite.de

1 Supplementary Figures and Tables

1.1 Supplementary Tables

Supplementary Table 1. Prominent function of identified mediators.

Mediator	Full name	Primary function	Reference
EGF	Epidermal growth factor	Stimulation of epidermal and dermal regeneration	(Zeng and Harris, 2014)
PDGF-AA/BB	Platelet-derived growth factor AA and BB	Embryo development and organogenesis	(Andrae et al., 2008)
HGF	Hepatocyte growth factor	Cell motility and morphogenesis	(Nakamura et al., 2011)
IP-10	Interferon gamma-induced protein 10	Attractant for T and NK cells	(Karin and Razon, 2018)
Eotaxin (CCL11)	Eosinophil chemotactic protein	Attractant for mast cells, macrophages, eosinophils, basophils, monocytes, DCs, NK cells, NK T cells, and activated T cells	(Pease and Horuk, 2009a, 2009b)
TARC	Thymus and activation regulated chemokine	Attractant for monocytes and Th2 T cells	(Pease and Horuk, 2009a, 2009b)
Angiopoietin-2		Vascular homeostasis	(Eklund et al., 2017)

1.2 Supplementary Figures

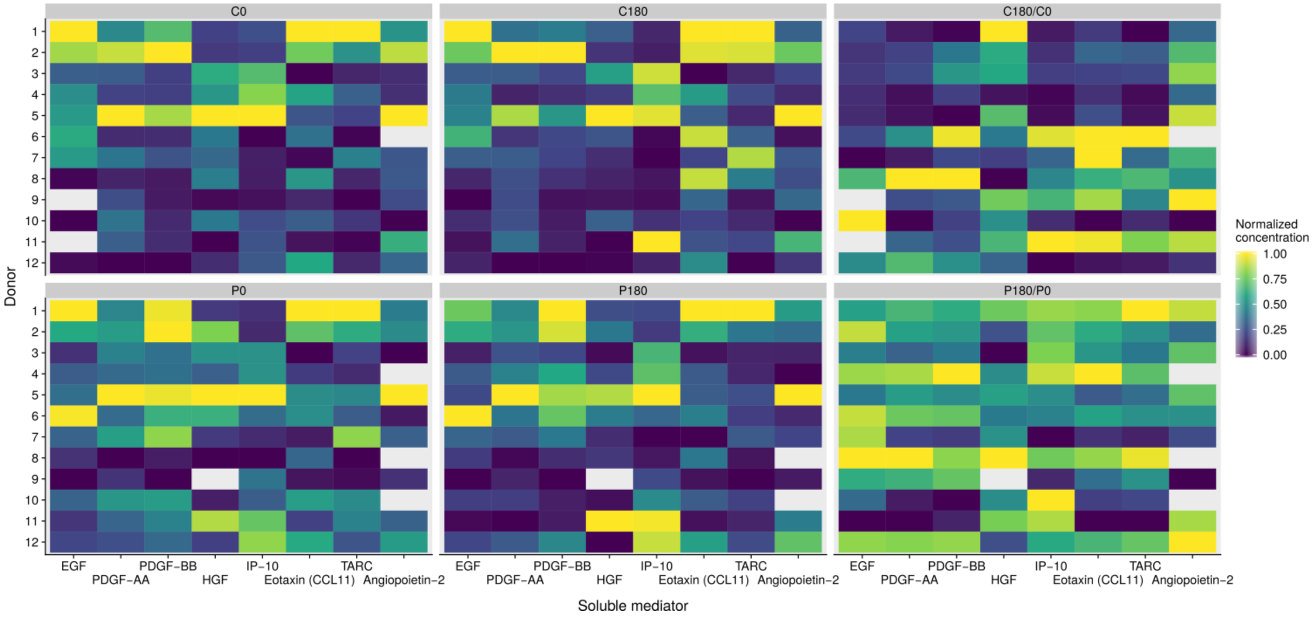

Supplementary Figure 1. Observed differences are not driven by a particular set of donors. Individual measurements for each donor and soluble mediator identified to change in the course of the parabolic flight. The measured concentrations and ratios were normalized to be in the range of 0 to 1. The normalization was performed for each soluble mediator and measurement time point or ratio. The normalization ensures that a donor with an all over high serum concentration of a soluble mediator would always have a value close to 1 and thus always have a bright color. Light gray indicate, that the concentration of mediator was below than the standard range.

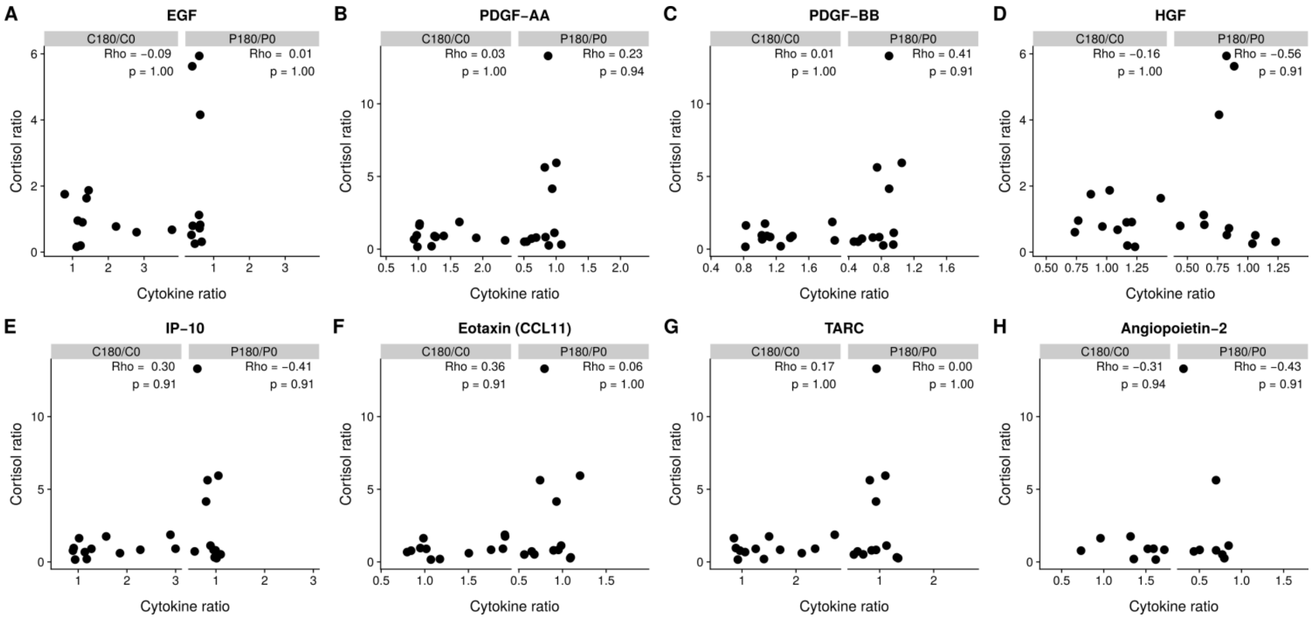

Supplementary Figure 2. Correlation of the rate change of cortisol levels to the rate change of the concentration of the soluble mediator. The association was measured using Spearman's correlation coefficient Rho. *p* indicate the probability of the observed correlation.

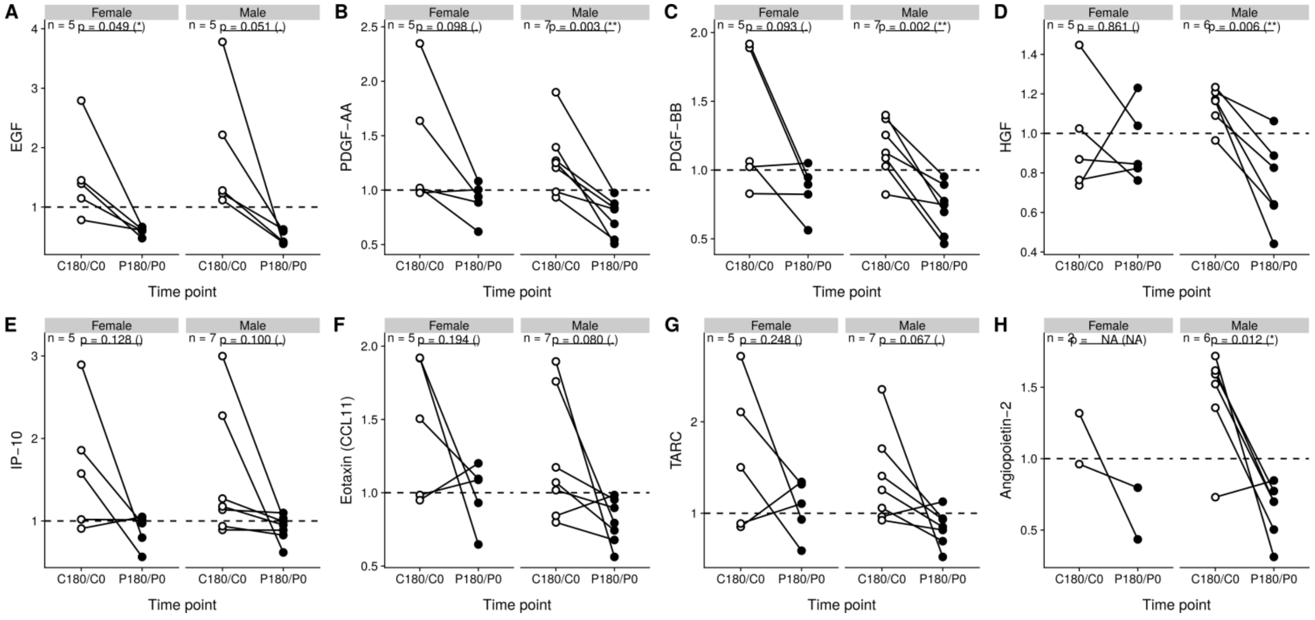

Supplementary Figure 3. Rate change of the concentration of the soluble mediators separated for females and males.

2 Supplementary References

Andrae, J., Gallini, R., and Betsholtz, C. (2008). Role of platelet-derived growth factors in physiology and medicine. *Genes Dev.* 22, 1276–1312. doi:10.1101/gad.1653708.

Eklund, L., Kangas, J., and Saharinen, P. (2017). Angiopoietin–Tie signalling in the cardiovascular and lymphatic systems. *Clin. Sci.* 131, 87–103. doi:10.1042/CS20160129.

Karin, N., and Razon, H. (2018). Chemokines beyond chemo-attraction: CXCL10 and its significant role in cancer and autoimmunity. *Cytokine* 109, 24–28. doi:10.1016/j.cyto.2018.02.012.

Nakamura, T., Sakai, K., Nakamura, T., and Matsumoto, K. (2011). Hepatocyte growth factor twenty years on: Much more than a growth factor. *J. Gastroenterol. Hepatol.* 26, 188–202. doi:10.1111/j.1440-1746.2010.06549.x.

Pease, J. E., and Horuk, R. (2009a). Chemokine receptor antagonists: Part 1. *Expert Opin. Ther. Pat.* 19, 39–58. doi:10.1517/13543770802641346.

Pease, J. E., and Horuk, R. (2009b). Chemokine receptor antagonists: Part 2. *Expert Opin. Ther. Pat.* 19, 199–221. doi:10.1517/13543770802641353.

Zeng, F., and Harris, R. C. (2014). Epidermal growth factor, from gene organization to bedside. *Semin. Cell Dev. Biol.* 0, 2–11. doi:10.1016/j.semcdb.2014.01.011.
